# CVD controlled growth of large-scale WS_2_ monolayers

**DOI:** 10.1039/c9ra06219j

**Published:** 2019-09-19

**Authors:** Zhuhua Xu, Yanfei Lv, Jingzhou Li, Feng Huang, Pengbo Nie, Siwei Zhang, Shichao Zhao, Shixi Zhao, Guodan Wei

**Affiliations:** College of Materials & Environmental Engineering, Hangzhou Dianzi University Hangzhou 310018 P. R. China zhaoshichao@hdu.edu.cn; Tsinghua-Berkeley Shenzhen Institute (TBSI), Tsinghua University Shenzhen 518055 P. R. China weiguodan@sz.tsinghua.edu.cn; Tsinghua Shenzhen International Graduate School, Tsinghua University Shenzhen 518000 P. R. China

## Abstract

Monolayer tungsten disulfide (WS_2_) with a direct band gap of *ca.* 2.0 eV and stable properties has been a hotspot in two-dimensional (2D) nanoelectronics and optoelectronics. However, it remains challenging to successfully prepare monolayer WS_2_. In this paper, we report the chemical vapor deposition (CVD) growth behavior of hexagonal WS_2_ monolayers by using WS_2_ powders and sodium triosulfate (Na_2_S_2_O_3_) as precursors. We observed the Na_2_S_2_O_3_ has a significant effect on the WS_2_ triangular and leaf-like shapes. In addition, based on proposed S-termination and W-termination theory, the growth mechanisms for different shapes of WS_2_ were discussed.

## Abstract 2

Monolayer tungsten disulfide (WS_2_) with a direct band gap of *ca.* 2.0 eV and stable properties has been a hotspot in two-dimensional (2D) nanoelectronics and optoelectronics.

## Graphical abstract

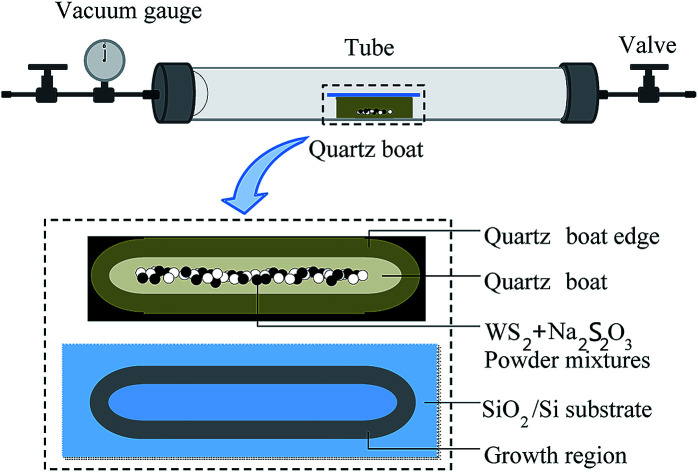

## 1. Introduction

In recent years, transition metal dichalcogenides with tunable band gap have attracted tremendous interest.^1^ Monolayer tungsten disulfide (WS_2_) with a direct band gap *ca.* 2.0 eV, shows potential applications in the fields of light emitting diodes, photodetectors, sensors, catalysts *etc.*^2–6^ Before the practical application, many efforts have been focused on the preparation. Approaches, such as physical and chemical exfoliation, chemical synthesis, atomic layer deposition, laser annealing, physical vapor deposition and chemical vapor deposition have been reported.^6–21^ Among them, exfoliation, chemical synthesis and chemical vapor deposition are the most used methods. Exfoliation is a versatile method for the low cost, scalable production of monolayer 2D materials.^6–10^ High crystal quality of monolayer WS_2_ prepared by this method is beneficial for fundamental property studies.^10^ Small size, nonuniform thickness, and agglomeration in solution are drawbacks of this method. Chemical synthesis, such as the reaction of H_3_PW_12_O_40_ with H_2_S, thermolysis of (NH_4_)WS_4_ and organometallic precursors, is facile but hard to obtain large-area film.^12–15^

Chemical vapor deposition (CVD) is an efficient, scalable method to grow large-scale monolayer WS_2_ film aiming for the fabrication of integrated device.^16,17^ Tungsten oxides (WO_3_), metallic tungsten, WCl_6_, W(CO)_6_, (NH_4_)_6_H_2_W_12_O_40_·*X*H_2_O, WS_2_ are used as tungsten precursor.^22–27^ The most utilized CVD process is the sulfurization of WO_3_ powders in sulfur vapor.^27–40^ However, the crystal quality was influenced by various experimental parameters, such as metal catalyst, pressure, substrate, the time of S-precursor introduction, temperature and location of precursor and substrate, carrier gas flow, pretreatment of the precursor or not, and growth temperature.^33,41–43^ The growth mechanism is unclear and under discussing. Wu *et al.* investigated the growth of MoS_2_ by sulfurization and found that sulfur enough condition promoted MoS_2_ film growth. They suggested that the film growth is determined by the background sulfur concentration.^44^ In our previous work, we also proposed that a delegation of tungsten precursor and an excess of sulfur are the predominate reasons for the monolayer growth.^2^ Cain *et al.* investigated the nucleation and they found that monolayer growth belongs to heterogeneous nucleation growth on oxi-chalcogenide nanoparticles.^45^ Therefore, the monolayer WS_2_ growth is complicated and mechanism is unclear. The CVD method needs to be further investigated.

To decrease the high melting point of the precursor should be considered before the controllable and reproducible CVD growth of WS_2_. To solve this problem, molten salt was introduced, such as sodium chloride (NaCl). Li *et al.* and Zhou *et al.* reported the alkali metal halides assisted growth of atomically thin metal dichalcogenides at relatively low temperature. Intermediate product volatile oxyhalides (as intermediate products) are considered as a promoter for monolayer growth.^46,47^ Modtland *et al.* prepared monolayer WS_2_ by chemical vapor transport deposition using WS_2_ as a precursor and NaCl as a transport agent. They suggested that nonvolatile WS_2_ reacts with NaCl to form gaseous tungsten chloride and sulfur.^48^ Wang *et al.* synthesized MoS_2_–WS_2_ in-plane heterostructures by ambient pressure chemical vapor deposition. They suggested Na-containing intermediate product is formed, which reduces the reaction energy.^49^ In addition, Lin *et al.* obtained 2H and 1T phase monolayer WS_2_ with assistance of iron oxide and NaCl.^50^ Shogo *et al.* prepared Nb-doped WS_2_ monolayers with the halide-assisted chemical vapor deposition (CVD).^51^

In this paper, we investigated the growth behavior of monolayer WS_2_ with WS_2_ powders and sodium triosulfate (Na_2_S_2_O_3_) as precursor in Ar/H_2_ atmosphere. Utilization of Na_2_S_2_O_3_ is based on the following consideration. Na_2_S_2_O_3_ differs from commonly used flux NaCl in that Na_2_S_2_O_3_ not only serves as the flux, but also the sulfur-rich precursor. Na_2_S_2_O_3_ will react with H_2_ to form excess H_2_S at high temperature, which can benefit the enhancement of WS_2_ crystal quality. Although, lots of work on the NaCl promoted growth of WS_2_ has been done, the role of sodium ions played is unclear.^52^ Here, we obtained separated triangular domains and continuous leaf-like film by CVD method at a relatively low temperature. In addition, we discussed the growth mechanism of leaf-like shape WS_2_ and the role of sodium salt. We believe our finds could provide a clue to prepare other 2D materials.

## 2. Experimental

### Monolayer WS_2_ growth

**Fig. 1:**
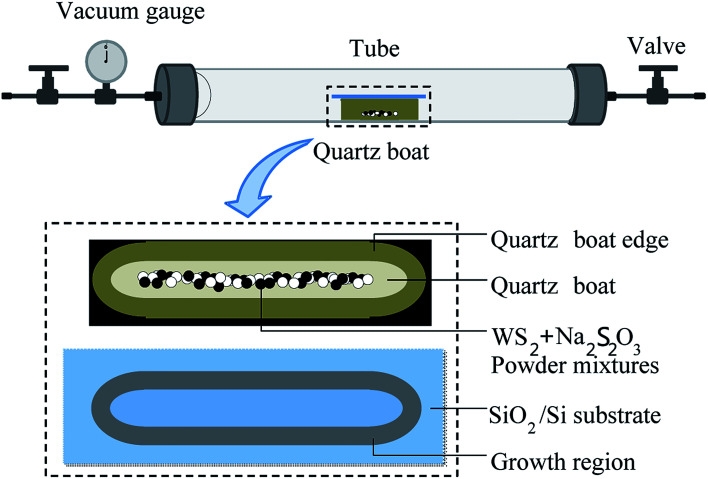
Schematic diagram of monolayer WS_2_ growth setup (upper), quartz boat and precursor (middle) and growth region (bottom) on SiO_2_/Si substrate. Growth zone shape is consistent with the edge of the quartz boat.

WS_2_ monolayer was prepared by co-firing WS_2_ powders and Na_2_S_2_O_3_ in a sealed tube. Fig. 1 shows growth setup consisting of a tube furnace and vacuum system. WS_2_ powders and solid Na_2_S_2_O_3_ were used as precursor. Silicon wafer with 300 nm of oxide layer (SiO_2_/Si) (100 × 15 mm) was used as the growth substrate. Before growth, the precursor (mixture of 0.1 g WS_2_ and 0.1 g Na_2_S_2_O_3_) was loaded into a quartz boat (80 × 10 mm) and placed in the center of the tube (25.4 mm in diameter). The substrate was covered on the quartz boat with the SiO_2_ side facing down to the precursor. For the growth of WS_2_ layers with triangular shape, the furnace tube was heated to 500 °C from room temperature in 40 min with 30 sccm Ar/H_2_ (5% H_2_). Then the tube was sealed at an atmospheric pressure and kept at 500 °C for 1 h before the tube was cooled to room temperature, naturally. For the growth of leaf-like WS_2_ film, the furnace tube temperature was 700 °C. Other experimental parameters kept unchanged.

### Characterization

Optical and photoluminescence (PL) imaging was taken on a Jiangnan MV3000 digital microscope and a Nib400 fluorescence microscope (Jiangnan Novel Optics Co., Ltd.), respectively. Scanning electron microscopy (SEM) was conducted on a desktop scanning electron microscope (COXEM EM-30 AX Plus). PL and Raman spectra were acquired on a home-built Raman system, consisting of an inverted microscope (Ti eclipse, Nikon), a Raman spectrometer (iHR320, Horiba) with a CCD camera (Syncerity, Horiba) and a semiconductor laser at 532 nm.

## 3. Results and discussion

**Fig. 2:**
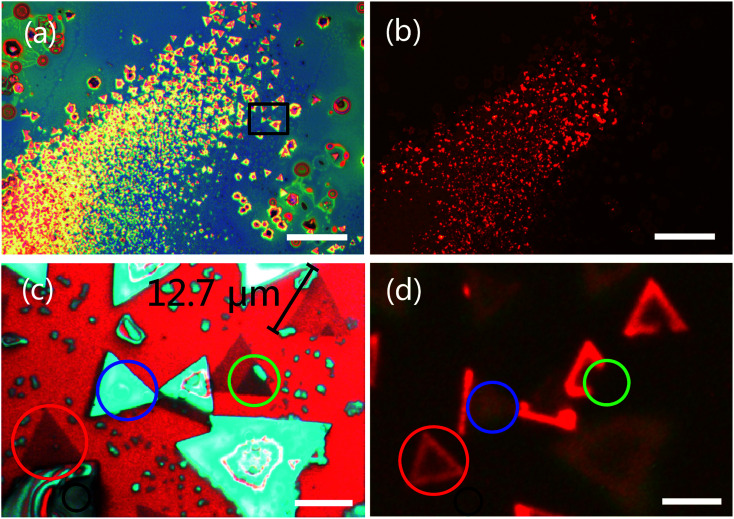
Optical microscopy (a) and photoluminescence (PL) (b) images of WS_2_ layers grown on SiO_2_/Si substrate. (c) Magnified optical microscopy and corresponding PL images taken from the boxed area in (a). (a) and (b) were taken at same location. The scale bars in figure (a) and (b) represent 100 μm, and 10 μm in (c) and (d). The red color in (b) and (d) is due to the PL emission of monolayer WS_2_.

**Fig. 3:**
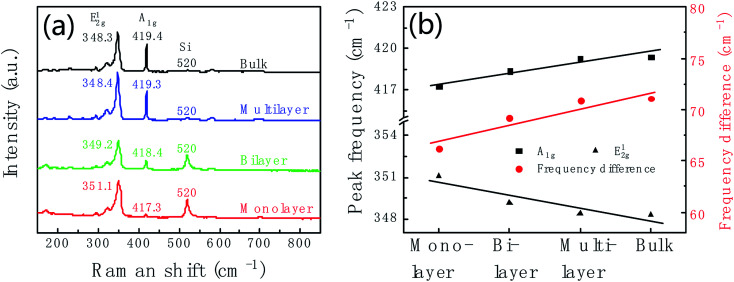
(a) Raman spectra of monolayer (red line), bilayer (green line), multilayer (blue line) and bulk (black line) WS_2_ corresponding to the sample in Fig. 2c. (b) Thickness dependent of Raman peak position and the peak frequency difference between E^1^_2g_ and A_1g_.

We observed the triangular WS_2_ crystals were grown on the substrate. Fig. 2a and c show optical microscopy image of WS_2_ layers with a triangular shape. Different contrast colors represent different thickness of WS_2_ (see discussion in Fig. 3). The triangular areas circled in black, red, green and blue colors are bulk, monolayer, bilayer and multilayer in Fig. 2c, respectively. The size of the monolayer WS_2_ domain is *ca.* 12.7 μm. The possible reason to why the domain cannot grow up is the low surface energy of the substrate. The hexagonal WS_2_ crystal can be assumed to begin from a hexagonal nucleus with three sides of W atom terminations and three sides of S atom terminations. When the W : S atomic ratio is less than 1 : 2, W terminations will grow faster than S terminations to become smaller or disappear until the hexagonal WS_2_ crystal grows to triangular in S rich structure. When the W : S atomic ratio is larger than 1 : 2, S terminations will grow faster than W terminations to become smaller or disappear until the hexagonal WS_2_ crystal grows to triangular in W rich structure. Both of these two cases could result in triangular shape WS_2_ growth. Therefore, the unbalanced the S and W termination growth at three sides of original hexagon could result in triangular WS_2_ growth.^53,54^ In this experiment, S source is sufficient from Na_2_S_2_O_3_, resulting in triangular WS_2_ growth.

Fig. 2b and d show PL images from monolayer WS_2_ with 485 nm excitation wavelengths. Being different from the indirect band gap of multilayer, monolayer has a direct band structure with high external quantum efficiency. Therefore, we only observed bright PL emission from monolayer WS_2_ with 485 nm excitation wavelengths in Fig. 2b and d. Clearly, the edge of the WS_2_ domain (circled in red color in Fig. 2d) shows intensive PL emission. Inhomogeneous PL intensity within a monolayer domain (circled in red color in Fig. 2d) is due to the uniformity of composition and defects from the center to the edge.^52^

Raman spectrum is commonly used to identify the layer number of WS_2_ layer. Fig. 3a shows Raman spectra of WS_2_ layers at room temperature with 532 nm laser excitation corresponding to the marked regions in Fig. 2c. The Raman data was calibrated by the Raman peak of Si at 520 cm^−1^. Two main peaks at 351.1 cm^−1^ and 417.3 cm^−1^ are characteristic Raman peaks of monolayer WS_2_. The peak at 351.1 cm^−1^ is due to the in-plane phonon mode E^1^_2g_, and 417.3 cm^−1^ for the out-of plane mode A_1g._ Therefore, the triangular domain circled in red color in Fig. 2c is monolayer WS_2_. The peak position and peak frequency difference between the two modes are the function of the number of WS_2_ layers. With the increase of the layer number, the E^1^_2g_ peak position decreases from 351.1 cm^−1^ of monolayer to 348.3 cm^−1^ of bulk; and the A_1g_ peak position increases from 417.3 cm^−1^ of monolayer to 419.4 cm^−1^ of bulk. The peak frequency difference increases with the increase of the thickness shown in Fig. 3b. The frequency differences are 66.2 cm^−1^, 69.2 cm^−1^, 70.9 cm^−1^, and 71.1 cm^−1^ corresponding to monolayer, bilayer, multilayer and bulk, respectively.

**Fig. 4:**
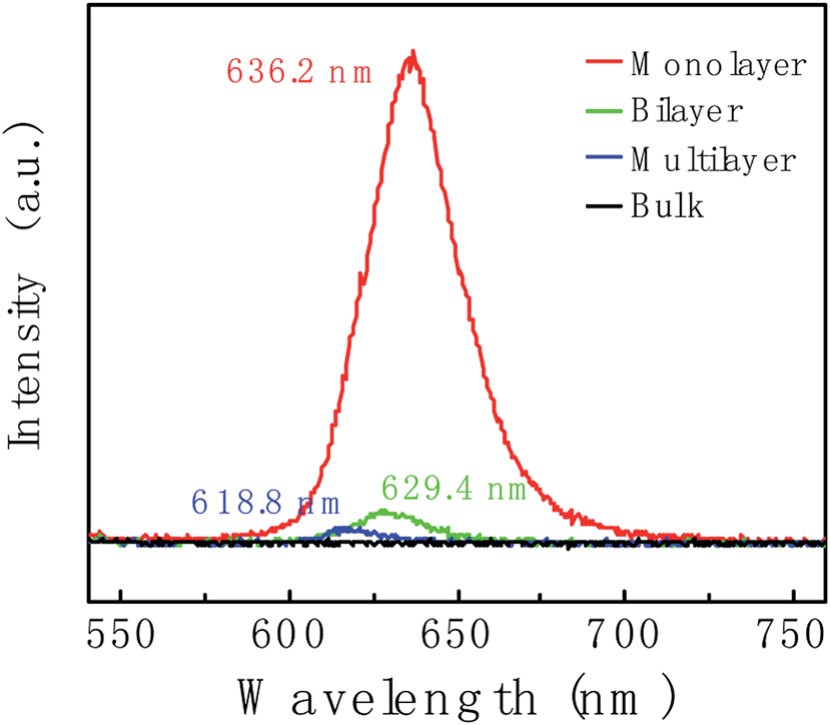
Photoluminescence (PL) spectra of WS_2_ layers corresponding to the region marked with a red circle, blue circle, green circle and black circle in Fig. 2c, respectively.

Fig. 4 shows the thickness-dependent PL spectra of WS_2_. An intense PL emission peak in the monolayer (red line) at 636.2 nm is observed, which is due to the A^−^ exciton emission. Compare to the monolayer, the PL intensity of bilayer (green line) is decreased by *ca.* 10-fold. For the multilayer (blue line), the PL intensity was further decreased. In bulk WS_2_ (black line), PL emission is hardly observed. Different behaviors in PL lie in the different band gap structures. Not like multilayer, monolayer has a direct band gap structure thus resulting in the intense PL emission. In addition, the PL peak position shows blueshift. We suspect the blueshift maybe be due to the defects and stress in the sample.^55,56^

**Fig. 5:**
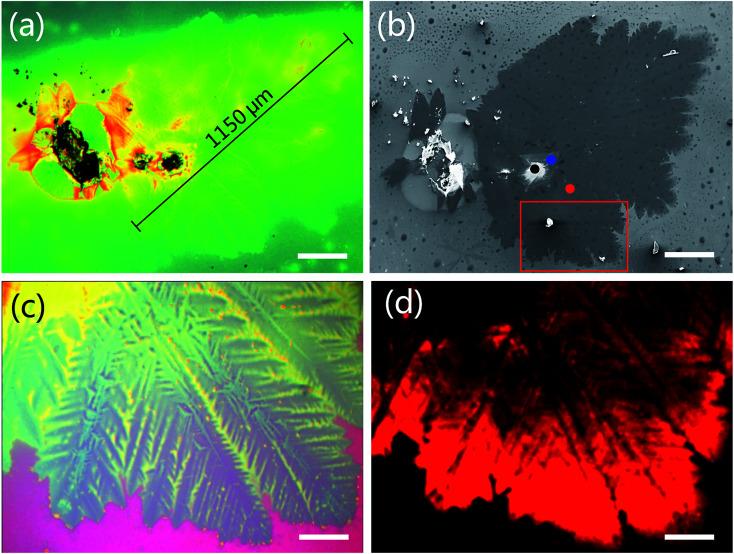
Optical microscopy (a) and SEM (b) images of WS_2_ film taken at the same location. (c) Magnified optical microscopy image of (a). (d) The photoluminescence (PL) image corresponding to the sample of (c). (c) and (d) are taken at the same location. The red color in (d) is due to the PL emission of monolayer WS_2_. The scale bars in figure (a), (b), (c) and (d) represent 200 μm, 200 μm, 50 μm and 50 μm, respectively.

**Fig. 6:**
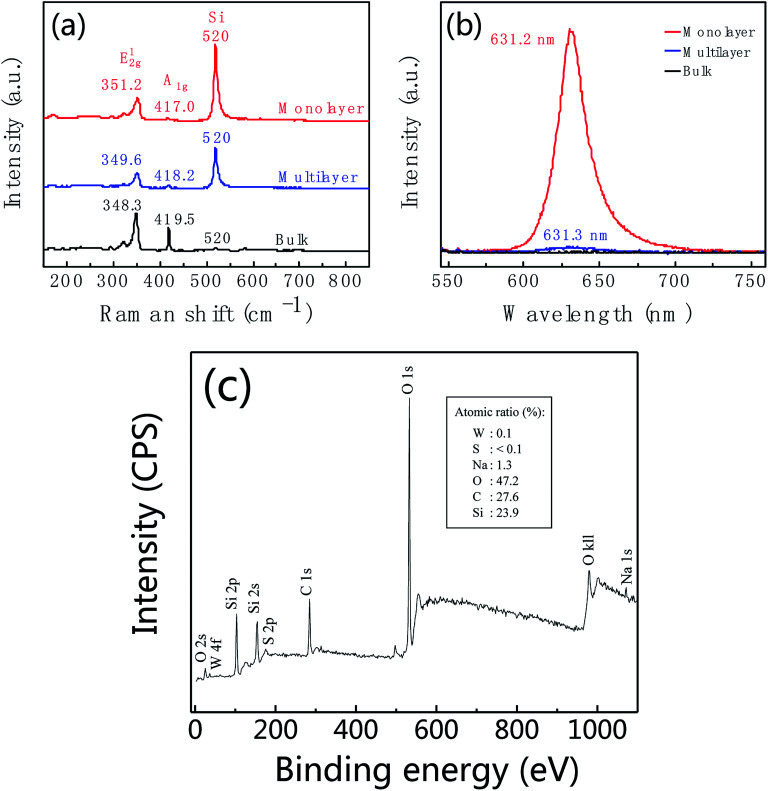
Raman (a) and photoluminescence (b) spectra of WS_2_ taken from the regions marked with red, blue and black spots in Fig. 5b. (c) XPS of leaf-like domain of as-prepared WS_2_. Insert is atomic ratio.

Besides the triangular WS_2_ domains, we also observed the leaf-like WS_2_ film in Fig. 5. Fig. 5a and c are the optical microscopy images of the leaf-like WS_2_ film. Fig. 5d is the PL image corresponding to the sample in Fig. 5c. The bright red color is due to the monolayer. The shape size is up to 1150 μm (Fig. 5a). Inside is bulk WS_2_ (marked with black dot in Fig. 5b). Around the bulk WS_2_ is multilayer WS_2_ (dark lines in Fig. 5d) extending in all direction like leaf veins. Continuous monolayer WS_2_ (red color in Fig. 5d) connects the multilayer. The thickness is determined by following Raman and PL data in Fig. 6. This unique interconnection of the WS_2_ domains will benefit the future large-scale application in optoelectronics such as photodetectors in which the monolayer of WS_2_ could capture the excitation light signal and the generated free carriers could transport through the “leaf veins” channels of the multilayer of WS_2_.

Fig. 6a and b shows the Raman and PL spectra of WS_2_ at different locations corresponding to the regions marked with red, blue and black spots in Fig. 5b. For the region marked with red color, Raman peaks of E^1^_2g_ and A_1g_ are at 351.2 cm^−1^ and 417.0 cm^−1^, respectively, with peak frequency difference of 65.8 cm^−1^ (red curve in Fig. 6a). As well as this region shows intensive PL emission at 631.2 nm. These features indicate that the region marked with a red dot in Fig. 5b is monolayer WS_2_. By further analysis, we found the regions marked with blue and black dots in Fig. 5b are multilayer and bulk, respectively. Fig. 6c is XPS of leaf-like domain showing W atomic ratio is 0.1% and S atomic ratio is <0.1%. Therefore, W : S atomic ratio is larger than 1 : 2. Carbon is from CO_2_ which was adsorbed on the surface of sample. Silicon is from substrate.

**Fig. 7:**
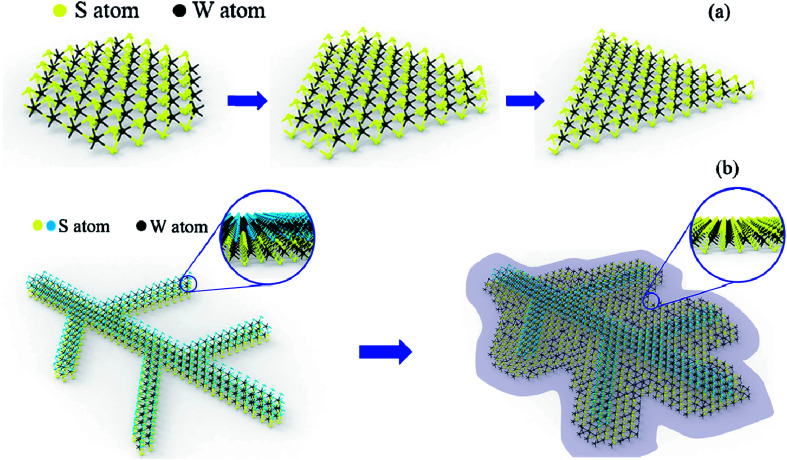
Schematic drawing of the growth mechanism of (a) triangular WS_2_ and (b) leaf-like WS_2_.

Here, the leaf-like shape WS_2_ growth mechanism should be different from the triangular WS_2_ and more like kinetically limited growth (Fig. 7). We suspect that the leaf-like shape is due to the preferential orientation growth. The above-mentioned triangle growth is due to preferential growth with W terminations. However, the preferential growth of leaf-like shape is S terminations. Sodium ions (Na^+^) from the sodium triosulfate (Na_2_S_2_O_3_) precursor is diffused all over the Si substrate during the heating up steps, wherein S terminations will absorb the Na^+^ to form sodium-passivated S terminations, resulting the terminated chemical reaction between S and W and the further growth of WS_2_ (Fig. 7a). At 500 °C, Na^+^ is physically adsorbed on substrate surface, so the absorption quantity is so little that there is no obvious effect on resulting sodium-passivated S termination. Thus, preferential growth of triangle shape is W terminations. At 700 °C, Na^+^ is chemically adsorbed on substrate surface, so the absorption quantity is enough to result sodium-passivated S termination. As a result, the preferential growth of leaf-like shape is S terminations (Fig. 7b) which is consistent with the XPS results that the W and S atomic ratio is larger than 1 : 2. The defects are introduced combined with the faster growth and lead to the multilayer growth with Stranski–Krastanow mode (SK mode) which refers to layer plus island growth: 2D film grows on the surface of substrate and there is distortion due to lattice mismatch between film and substrate. Then, the 2D film will adsorb the deposited atoms, forming islands in the way of nucleation growth, and eventually grows into film. Therefore, the “leaf veins” (in Fig. 5c and d) are produced. Followed the growth of “leaf veins”, is the relatively slow-growing S terminations as shown in the inset of Fig. 7b, where triangular shape of WS_2_ could be formed to successfully fill the gaps among the branched leaf veins. Therefore, the interconnected WS_2_ in large scale has been formed.

## 4. Conclusion

In conclusion, we have successfully prepared WS_2_ layers by CVD with the WS_2_ and Na_2_S_2_O_3_ as precursors. Na_2_S_2_O_3_ used as sulfur precursor and molten salt has a significant effect on the film growth. Triangular and leaf-like shapes of WS_2_ film were obtained. The S-termination could absorb the Na^+^ to form sodium-passivated S terminations and terminate the further growth of WS_2_, resulting the triangular shape of WS_2_. On contrast, the W-termination grows much faster than the S-termination, resulting the leaf vein shape growth of WS_2_. Followed the growth of “leaf veins”, there are more locations of S-termination in the branched veins of WS_2_ which is favorable for triangular WS_2_ growth to fill the gaps among the various veins in the leaf structured WS_2_. Therefore, the controllable WS_2_ layers have been demonstrated with Na_2_S_2_O_3_ precursors as growth motivators which could provide informative guidance for future large-scale 2D growth.

## Conflicts of interest

There are no conflicts of interest to declare.

## Supplementary Material
